# 1-[6-(Hydroxy­meth­yl)-2-pyrid­yl]-3-(2,4,6-trimethyl­benz­yl)-1*H*-imidazol-3-ium bromide

**DOI:** 10.1107/S1600536808034764

**Published:** 2008-10-31

**Authors:** Chuang Zhou, Xiang-Ge Zhou, Yu-Ping Qiu, Mei-Ming Luo

**Affiliations:** a Key Laboratory of Green Chemistry and Technology of Ministry of Education, College of Chemistry, Sichuan University, Chengdu 610064, People’s Republic of China

## Abstract

In the title compound, C_19_H_22_N_3_O^+^·Br^−^, the imidazole ring is approximately coplanar with the pyridine ring [dihedral angle = 0.88 (13)°] and nearly perpendicular to the benzene ring [dihedral angle = 81.70 (13)°]. O—H⋯Br and C—H⋯Br hydrogen bonding helps to stabilize the crystal structure.

## Related literature

For general background, see: Liddle *et al.* (2007 ▶); Ren *et al.* (2007 ▶); Arnold & Wilson (2007 ▶); Chianese & Crabtree (2005 ▶); Dyson *et al.* (2008 ▶); Patel *et al.* (2006 ▶). For synthesis, see: Hosseinzadeh *et al.* (2006 ▶).
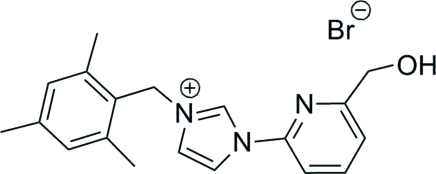


## Experimental

### 

#### Crystal data

C_19_H_22_N_3_O^+^·Br^−^
*M* *_r_* = 388.31
Monoclinic, 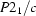

*a* = 11.2315 (3) Å
*b* = 11.5390 (3) Å
*c* = 14.3673 (4) Å
β = 100.833 (2)°
*V* = 1828.82 (9) Å^3^
*Z* = 4
Mo *K*α radiation
μ = 2.26 mm^−1^
*T* = 296 (2) K
0.50 × 0.48 × 0.40 mm

#### Data collection

Bruker SMART CCD area-detector diffractometer
Absorption correction: multi-scan (*SADABS*; Sheldrick, 1996 ▶) *T*_min_ = 0.320, *T*_max_ = 0.405
13818 measured reflections
4184 independent reflections
2287 reflections with *I* > 2σ(*I*)
*R*_int_ = 0.048

#### Refinement

*R*[*F* ^2^ > 2σ(*F* ^2^)] = 0.044
*wR*(*F* ^2^) = 0.116
*S* = 1.01
4184 reflections
220 parameters
H-atom parameters constrained
Δρ_max_ = 0.41 e Å^−3^
Δρ_min_ = −0.60 e Å^−3^

### 

Data collection: *SMART* (Bruker, 1997 ▶); cell refinement: *SAINT* (Bruker, 1997 ▶); data reduction: *SAINT*; program(s) used to solve structure: *SHELXS97* (Sheldrick, 2008 ▶); program(s) used to refine structure: *SHELXL97* (Sheldrick, 2008 ▶); molecular graphics: *ORTEP-3 for Windows* (Farrugia, 1997 ▶); software used to prepare material for publication: *WinGX* (Farrugia, 1999 ▶).

## Supplementary Material

Crystal structure: contains datablocks global, I. DOI: 10.1107/S1600536808034764/xu2459sup1.cif

Structure factors: contains datablocks I. DOI: 10.1107/S1600536808034764/xu2459Isup2.hkl

Additional supplementary materials:  crystallographic information; 3D view; checkCIF report

## Figures and Tables

**Table 1:** Hydrogen-bond geometry (Å, °)

*D*—H⋯*A*	*D*—H	H⋯*A*	*D*⋯*A*	*D*—H⋯*A*
O1—H1*A*⋯Br1	0.82	2.49	3.227 (2)	151
C7—H7*A*⋯Br1^i^	0.93	2.75	3.598 (2)	152
C8—H8*A*⋯Br1^ii^	0.93	2.89	3.745 (2)	154
C10—H10*B*⋯Br1^i^	0.97	2.91	3.813 (2)	155

Symmetry codes: (i) 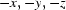; (ii) 
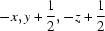
.

**Fig. 1.**
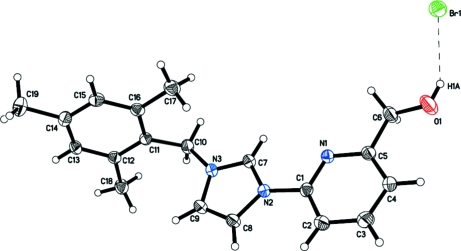
The molecular structure of the title compound with displacement ellipsoids drawn at the 30% probability level. Dashed line indicates the O—H···Br hydrogen bonding.

## supplementary crystallographic information

### Comment

The range of N-heterocyclic carbenes (NHCs) is expanding rapidly since many homogeneous catalysts rely on NHC-based supporting ligands for steric and electronic control. Recently the study of [C, O] chelating NHC ligands have attracted increasing attention. The bonding between the NHC and metal can be enhanced by alkoxide or phenoxide as a sidearm through the incorporation of chelating anionic oxygen (Liddle *et al.*, 2007; Ren *et al.*, 2007). These kind of ligands are of great significance to early metal catalysis and carbene chemistry (Arnold & Wilson, 2007; Chianese & Crabtree, 2005; Dyson *et al.*, 2008; Patel *et al.*, 2006). The title compound, a stable precursor imidazolium salt of a tridentate alkoxide-functionalized NHC ligands, was synthesized in moderate yield by reacting [6-(1*H*-imidazol-1-yl)pyridin-2-yl]methanol with 2-(bromomethyl)-1,3,5-trimethylbenzene in acetonitrile.

In the title compound (Fig. 1), the pyridine and imidazole rings are coplanar, the dihedral angle between the plane of the pyridine ring and the plane of the imidazole ring is 0.88°. In addition, the dihedral angle between the imidazole ring and the benzene ring is 81.70 °. This might be a result of intermolecular O—H···Br interactions and steric effects. The O—H···Br and C—H···Br hydrogen bonding (Table 1) helps to stabilize the crystal structure.

### Experimental

[6-(1*H*-Imidazol-1-yl)pyridin-2-yl]methanol was prepared with the reported methods (Hosseinzadeh *et al.*, 2006). The title compound was synthesized by dissolving [6-(1*H*-imidazol-1-yl)pyridin-2-yl]methanol (0.35 g, 2.0 mmol) and 2-(bromomethyl)-1,3,5-trimethylbenzene (0.85 g, 4.0 mmol) in 10 ml of acetonitrile. The mixture was stirred at 333 K for 15 h and the resulting precipitate was filtered, washed with ether. After removal of the solvent in vacuo the off-white crude product was purified by flash chromatography (CH_2_Cl_2_/CH_3_OH (5/1, *v*/*v*)) to afford the product as a white solid (0.60 g, 77%). Colorless single crystals suitable for X-ray diffraction were obtained at ambient temperature by slow evaporation of a CH_2_Cl_2_/CH_3_OH solution (5/1, *v*/*v*)) over a period of several days.

### Refinement

All H atom were positioned geometrically with C—H = 0.93 Å (aromatic) or 0.96 Å (methyl) and O—H = 0.82 Å, and refined using a riding model with 1.5*U*_eq_(C) for methyl and *U*_iso_(H) = 1.2*U*_eq_(C,*O*) for others.

### Crystal data

**Table:**

C_19_H_22_N_3_O^+^·Br^−^	*F*(000) = 800
*M**_r_* = 388.31	*D*_x_ = 1.410 Mg m^−^^3^
Monoclinic, *P*2_1_/*c*	Mo *K*α radiation, λ = 0.71073 Å
Hall symbol: -P 2ybc	Cell parameters from 3980 reflections
*a* = 11.2315 (3) Å	θ = 2.8–26.7°
*b* = 11.5390 (3) Å	µ = 2.26 mm^−^^1^
*c* = 14.3673 (4) Å	*T* = 296 K
β = 100.833 (2)°	Block, colourless
*V* = 1828.82 (9) Å^3^	0.50 × 0.48 × 0.40 mm
*Z* = 4	

### Data collection

**Table:**

Bruker SAMRT CCD area-detector diffractometer	4184 independent reflections
Radiation source: fine-focus sealed tube	2287 reflections with *I* > 2σ(*I*)
graphite	*R*_int_ = 0.048
φ and ω scans	θ_max_ = 27.5°, θ_min_ = 1.9°
Absorption correction: multi-scan (SADABS; Sheldrick, 1996)	*h* = −14→14
*T*_min_ = 0.320, *T*_max_ = 0.405	*k* = −13→14
13818 measured reflections	*l* = −17→18

### Refinement

**Table:**

Refinement on *F*^2^	Primary atom site location: structure-invariant direct methods
Least-squares matrix: full	Secondary atom site location: difference Fourier map
*R*[*F*^2^ > 2σ(*F*^2^)] = 0.044	Hydrogen site location: inferred from neighbouring sites
*wR*(*F*^2^) = 0.116	H-atom parameters constrained
*S* = 1.01	*w* = 1/[σ^2^(*F*_o_^2^) + (0.05*P*)^2^ + 0.57*P*] where *P* = (*F*_o_^2^ + 2*F*_c_^2^)/3
4184 reflections	(Δ/σ)_max_ < 0.001
220 parameters	Δρ_max_ = 0.41 e Å^−^^3^
0 restraints	Δρ_min_ = −0.60 e Å^−^^3^

### Special details

**Table:**

Geometry. All e.s.d.'s (except the e.s.d. in the dihedral angle between two l.s. planes) are estimated using the full covariance matrix. The cell e.s.d.'s are taken into account individually in the estimation of e.s.d.'s in distances, angles and torsion angles; correlations between e.s.d.'s in cell parameters are only used when they are defined by crystal symmetry. An approximate (isotropic) treatment of cell e.s.d.'s is used for estimating e.s.d.'s involving l.s. planes.
Refinement. Refinement of *F*^2^ against ALL reflections. The weighted *R*-factor *wR* and goodness of fit *S* are based on *F*^2^, conventional *R*-factors *R* are based on *F*, with *F* set to zero for negative *F*^2^. The threshold expression of *F*^2^ > σ(*F*^2^) is used only for calculating *R*-factors(gt) *etc*. and is not relevant to the choice of reflections for refinement. *R*-factors based on *F*^2^ are statistically about twice as large as those based on *F*, and *R*- factors based on ALL data will be even larger.

### Fractional atomic coordinates and isotropic or equivalent isotropic displacement parameters (Å^2^)

**Table:**

	*x*	*y*	*z*	*U*_iso_*/*U*_eq_	
Br1	−0.28302 (3)	0.00291 (3)	−0.05646 (2)	0.05606 (10)	
O1	−0.21350 (16)	0.2315 (2)	0.07160 (15)	0.0662 (7)	
H1A	−0.2478	0.1920	0.0271	0.099*	
N1	0.09397 (17)	0.27426 (17)	0.18365 (14)	0.0321 (5)	
N2	0.28135 (17)	0.30337 (16)	0.27893 (14)	0.0305 (5)	
N3	0.44633 (17)	0.20953 (17)	0.27441 (14)	0.0334 (5)	
C1	0.1546 (2)	0.3348 (2)	0.25443 (17)	0.0316 (6)	
C2	0.1075 (2)	0.4190 (2)	0.3045 (2)	0.0445 (8)	
H2A	0.1547	0.4574	0.3553	0.053*	
C3	−0.0135 (3)	0.4434 (3)	0.2752 (2)	0.0531 (9)	
H3A	−0.0507	0.4989	0.3068	0.064*	
C4	−0.0795 (2)	0.3852 (2)	0.1986 (2)	0.0444 (8)	
H4A	−0.1606	0.4034	0.1764	0.053*	
C5	−0.0234 (2)	0.2998 (2)	0.15572 (18)	0.0356 (7)	
C6	−0.0886 (2)	0.2276 (2)	0.0752 (2)	0.0445 (8)	
H6A	−0.0703	0.2563	0.0160	0.053*	
H6B	−0.0608	0.1480	0.0832	0.053*	
C7	0.3316 (2)	0.2205 (2)	0.23424 (18)	0.0346 (6)	
H7A	0.2919	0.1775	0.1830	0.042*	
C8	0.3694 (2)	0.3481 (2)	0.34984 (18)	0.0380 (7)	
H8A	0.3598	0.4075	0.3916	0.046*	
C9	0.4719 (2)	0.2892 (2)	0.34690 (18)	0.0384 (7)	
H9A	0.5466	0.3002	0.3866	0.046*	
C10	0.5327 (2)	0.1277 (2)	0.24373 (18)	0.0376 (7)	
H10A	0.5877	0.1707	0.2121	0.045*	
H10B	0.4880	0.0740	0.1982	0.045*	
C11	0.6055 (2)	0.0601 (2)	0.32432 (17)	0.0312 (6)	
C12	0.7292 (2)	0.0816 (2)	0.35423 (17)	0.0320 (6)	
C13	0.7950 (2)	0.0118 (2)	0.42336 (17)	0.0339 (6)	
H13A	0.8775	0.0261	0.4427	0.041*	
C14	0.7435 (2)	−0.0781 (2)	0.46487 (18)	0.0368 (7)	
C15	0.6197 (2)	−0.0969 (2)	0.43645 (18)	0.0370 (7)	
H15A	0.5830	−0.1563	0.4645	0.044*	
C16	0.5498 (2)	−0.0293 (2)	0.36734 (18)	0.0336 (7)	
C17	0.4158 (2)	−0.0533 (3)	0.3403 (2)	0.0470 (8)	
H17A	0.3971	−0.1241	0.3696	0.070*	
H17B	0.3935	−0.0604	0.2727	0.070*	
H17C	0.3713	0.0094	0.3615	0.070*	
C18	0.7936 (2)	0.1776 (2)	0.3128 (2)	0.0466 (8)	
H18A	0.8792	0.1721	0.3367	0.070*	
H18B	0.7643	0.2511	0.3303	0.070*	
H18C	0.7783	0.1708	0.2450	0.070*	
C19	0.8180 (3)	−0.1551 (3)	0.5387 (2)	0.0521 (8)	
H19A	0.9026	−0.1385	0.5423	0.078*	
H19B	0.8026	−0.2348	0.5215	0.078*	
H19C	0.7963	−0.1408	0.5992	0.078*	

### Atomic displacement parameters (Å^2^)

**Table:**

	*U*^11^	*U*^22^	*U*^33^	*U*^12^	*U*^13^	*U*^23^
Br1	0.0685 (2)	0.04819 (17)	0.04329 (16)	−0.00279 (17)	−0.01057 (14)	−0.00348 (15)
O1	0.0395 (11)	0.0820 (15)	0.0724 (15)	−0.0069 (11)	−0.0020 (11)	−0.0294 (12)
N1	0.0311 (11)	0.0305 (11)	0.0330 (12)	−0.0008 (9)	0.0022 (9)	0.0017 (9)
N2	0.0304 (11)	0.0302 (11)	0.0296 (11)	0.0007 (9)	0.0021 (9)	0.0001 (9)
N3	0.0290 (11)	0.0338 (11)	0.0346 (12)	0.0002 (9)	−0.0014 (9)	0.0000 (9)
C1	0.0312 (13)	0.0309 (13)	0.0324 (14)	0.0006 (11)	0.0049 (11)	0.0053 (11)
C2	0.0388 (15)	0.0424 (15)	0.0495 (17)	0.0016 (13)	0.0011 (13)	−0.0122 (13)
C3	0.0433 (17)	0.0473 (16)	0.068 (2)	0.0062 (14)	0.0089 (15)	−0.0158 (16)
C4	0.0314 (14)	0.0379 (15)	0.0611 (19)	0.0021 (12)	0.0015 (13)	−0.0063 (14)
C5	0.0373 (14)	0.0316 (13)	0.0365 (15)	−0.0057 (11)	0.0031 (12)	0.0074 (11)
C6	0.0367 (15)	0.0509 (17)	0.0420 (16)	−0.0047 (13)	−0.0029 (13)	−0.0032 (14)
C7	0.0336 (14)	0.0359 (14)	0.0319 (14)	−0.0010 (12)	−0.0001 (11)	−0.0012 (12)
C8	0.0393 (14)	0.0377 (14)	0.0341 (15)	−0.0042 (12)	0.0000 (12)	−0.0040 (12)
C9	0.0355 (14)	0.0402 (15)	0.0360 (15)	−0.0055 (12)	−0.0023 (12)	−0.0051 (12)
C10	0.0335 (14)	0.0437 (15)	0.0342 (15)	0.0039 (12)	0.0030 (12)	−0.0027 (12)
C11	0.0303 (13)	0.0309 (13)	0.0317 (14)	0.0014 (11)	0.0042 (11)	−0.0022 (11)
C12	0.0311 (13)	0.0320 (13)	0.0336 (14)	−0.0019 (11)	0.0077 (11)	−0.0022 (11)
C13	0.0246 (12)	0.0404 (15)	0.0356 (13)	−0.0004 (12)	0.0031 (10)	−0.0022 (12)
C14	0.0416 (15)	0.0386 (14)	0.0306 (14)	0.0082 (12)	0.0073 (12)	0.0002 (12)
C15	0.0408 (14)	0.0341 (14)	0.0383 (15)	−0.0060 (12)	0.0131 (12)	0.0037 (12)
C16	0.0325 (13)	0.0332 (14)	0.0359 (14)	−0.0008 (11)	0.0087 (11)	−0.0080 (11)
C17	0.0353 (15)	0.0474 (16)	0.0582 (19)	−0.0060 (13)	0.0088 (14)	−0.0031 (15)
C18	0.0364 (15)	0.0491 (17)	0.0519 (18)	−0.0067 (13)	0.0018 (13)	0.0090 (14)
C19	0.0507 (17)	0.0587 (18)	0.0465 (18)	0.0092 (15)	0.0079 (14)	0.0163 (15)

### Geometric parameters (Å, °)

**Table:**

O1—C6	1.394 (3)	C10—C11	1.504 (3)
O1—H1A	0.8200	C10—H10A	0.9700
N1—C1	1.314 (3)	C10—H10B	0.9700
N1—C5	1.336 (3)	C11—C12	1.398 (3)
N2—C7	1.335 (3)	C11—C16	1.408 (4)
N2—C8	1.380 (3)	C12—C13	1.380 (3)
N2—C1	1.447 (3)	C12—C18	1.505 (4)
N3—C7	1.315 (3)	C13—C14	1.377 (4)
N3—C9	1.378 (3)	C13—H13A	0.9300
N3—C10	1.479 (3)	C14—C15	1.390 (3)
C1—C2	1.372 (4)	C14—C19	1.510 (4)
C2—C3	1.373 (4)	C15—C16	1.385 (3)
C2—H2A	0.9300	C15—H15A	0.9300
C3—C4	1.380 (4)	C16—C17	1.509 (3)
C3—H3A	0.9300	C17—H17A	0.9600
C4—C5	1.377 (4)	C17—H17B	0.9600
C4—H4A	0.9300	C17—H17C	0.9600
C5—C6	1.500 (3)	C18—H18A	0.9600
C6—H6A	0.9700	C18—H18B	0.9600
C6—H6B	0.9700	C18—H18C	0.9600
C7—H7A	0.9300	C19—H19A	0.9600
C8—C9	1.344 (3)	C19—H19B	0.9600
C8—H8A	0.9300	C19—H19C	0.9600
C9—H9A	0.9300		
			
C6—O1—H1A	109.5	C11—C10—H10A	108.9
C1—N1—C5	117.0 (2)	N3—C10—H10B	108.9
C7—N2—C8	108.3 (2)	C11—C10—H10B	108.9
C7—N2—C1	123.3 (2)	H10A—C10—H10B	107.8
C8—N2—C1	128.4 (2)	C12—C11—C16	119.5 (2)
C7—N3—C9	108.4 (2)	C12—C11—C10	120.7 (2)
C7—N3—C10	125.0 (2)	C16—C11—C10	119.8 (2)
C9—N3—C10	126.6 (2)	C13—C12—C11	119.0 (2)
N1—C1—C2	126.0 (2)	C13—C12—C18	118.8 (2)
N1—C1—N2	113.4 (2)	C11—C12—C18	122.2 (2)
C2—C1—N2	120.6 (2)	C14—C13—C12	122.6 (2)
C1—C2—C3	116.2 (2)	C14—C13—H13A	118.7
C1—C2—H2A	121.9	C12—C13—H13A	118.7
C3—C2—H2A	121.9	C13—C14—C15	117.9 (2)
C2—C3—C4	119.8 (3)	C13—C14—C19	121.6 (2)
C2—C3—H3A	120.1	C15—C14—C19	120.4 (2)
C4—C3—H3A	120.1	C16—C15—C14	121.5 (2)
C5—C4—C3	118.8 (3)	C16—C15—H15A	119.2
C5—C4—H4A	120.6	C14—C15—H15A	119.2
C3—C4—H4A	120.6	C15—C16—C11	119.3 (2)
N1—C5—C4	122.1 (2)	C15—C16—C17	119.0 (2)
N1—C5—C6	114.9 (2)	C11—C16—C17	121.7 (2)
C4—C5—C6	123.0 (2)	C16—C17—H17A	109.5
O1—C6—C5	110.7 (2)	C16—C17—H17B	109.5
O1—C6—H6A	109.5	H17A—C17—H17B	109.5
C5—C6—H6A	109.5	C16—C17—H17C	109.5
O1—C6—H6B	109.5	H17A—C17—H17C	109.5
C5—C6—H6B	109.5	H17B—C17—H17C	109.5
H6A—C6—H6B	108.1	C12—C18—H18A	109.5
N3—C7—N2	109.0 (2)	C12—C18—H18B	109.5
N3—C7—H7A	125.5	H18A—C18—H18B	109.5
N2—C7—H7A	125.5	C12—C18—H18C	109.5
C9—C8—N2	106.7 (2)	H18A—C18—H18C	109.5
C9—C8—H8A	126.7	H18B—C18—H18C	109.5
N2—C8—H8A	126.7	C14—C19—H19A	109.5
C8—C9—N3	107.6 (2)	C14—C19—H19B	109.5
C8—C9—H9A	126.2	H19A—C19—H19B	109.5
N3—C9—H9A	126.2	C14—C19—H19C	109.5
N3—C10—C11	113.2 (2)	H19A—C19—H19C	109.5
N3—C10—H10A	108.9	H19B—C19—H19C	109.5
			
C5—N1—C1—C2	2.5 (4)	C7—N3—C9—C8	0.4 (3)
C5—N1—C1—N2	−178.8 (2)	C10—N3—C9—C8	178.4 (2)
C7—N2—C1—N1	−0.3 (3)	C7—N3—C10—C11	−132.4 (2)
C8—N2—C1—N1	−179.0 (2)	C9—N3—C10—C11	49.8 (3)
C7—N2—C1—C2	178.5 (2)	N3—C10—C11—C12	−109.5 (3)
C8—N2—C1—C2	−0.2 (4)	N3—C10—C11—C16	73.4 (3)
N1—C1—C2—C3	−2.0 (4)	C16—C11—C12—C13	2.1 (4)
N2—C1—C2—C3	179.4 (2)	C10—C11—C12—C13	−175.0 (2)
C1—C2—C3—C4	−0.8 (4)	C16—C11—C12—C18	−178.5 (2)
C2—C3—C4—C5	2.8 (4)	C10—C11—C12—C18	4.4 (4)
C1—N1—C5—C4	−0.2 (4)	C11—C12—C13—C14	−0.5 (4)
C1—N1—C5—C6	−179.6 (2)	C18—C12—C13—C14	−179.8 (2)
C3—C4—C5—N1	−2.4 (4)	C12—C13—C14—C15	−1.2 (4)
C3—C4—C5—C6	177.0 (3)	C12—C13—C14—C19	178.5 (2)
N1—C5—C6—O1	160.1 (2)	C13—C14—C15—C16	1.2 (4)
C4—C5—C6—O1	−19.3 (4)	C19—C14—C15—C16	−178.5 (3)
C9—N3—C7—N2	−0.9 (3)	C14—C15—C16—C11	0.4 (4)
C10—N3—C7—N2	−179.0 (2)	C14—C15—C16—C17	−179.4 (2)
C8—N2—C7—N3	1.0 (3)	C12—C11—C16—C15	−2.1 (4)
C1—N2—C7—N3	−177.9 (2)	C10—C11—C16—C15	175.0 (2)
C7—N2—C8—C9	−0.8 (3)	C12—C11—C16—C17	177.7 (2)
C1—N2—C8—C9	178.1 (2)	C10—C11—C16—C17	−5.2 (4)
N2—C8—C9—N3	0.2 (3)		

### Hydrogen-bond geometry (Å, °)

**Table:**

*D*—H···*A*	*D*—H	H···*A*	*D*···*A*	*D*—H···*A*
O1—H1A···Br1	0.82	2.49	3.227 (2)	151
C7—H7A···Br1^i^	0.93	2.75	3.598 (2)	152
C8—H8A···Br1^ii^	0.93	2.89	3.745 (2)	154
C10—H10B···Br1^i^	0.97	2.91	3.813 (2)	155

Symmetry codes: (i) −*x*, −*y*, −*z*; (ii) −*x*, *y*+1/2, −*z*+1/2.
